# Predictive value analysis of the interaction network of Tks4 scaffold protein in colon cancer

**DOI:** 10.3389/fmolb.2024.1414805

**Published:** 2024-08-21

**Authors:** Álmos Tilajka, Anita Kurilla, Loretta László, Anna Lovrics, Julianna Novák, Tamás Takács, László Buday, Virag Vas

**Affiliations:** ^1^ Institute of Molecular Life Sciences, HUN-REN Research Centre for Natural Sciences, Budapest, Hungary; ^2^ Doctoral School of Biology, Institute of Biology, ELTE Eötvös Loránd University, Budapest, Hungary; ^3^ Department of Molecular Biology, Semmelweis University, Budapest, Hungary

**Keywords:** colon cancer, biomarker research, Tks4, cortactin, SRC, N-wasp, CD2AP, GRB2

## Abstract

**Background:**

Colorectal carcinoma (CRC) has emerged as one of the most widespread cancers and was the third leading cause of cancer-related mortality in 2020. The role of the podosomal protein Tks4 in tumor formation and progression is well established, including its involvement in gastric carcinoma and hepatocellular carcinoma; however, exploration of Tks4 and its associated EMT-regulating interactome in the context of colon cancer remains largely unexplored.

**Methods:**

We conducted a comprehensive bioinformatic analysis to investigate the mRNA and protein expression levels of Tks4 and its associated partner molecules (CD2AP, GRB2, WASL, SRC, CTTN, and CAPZA1) across different tumor types. We quantified the expression levels of Tks4 and its partner molecules using qPCR, utilizing a TissueScan colon cancer array. We then validated the usefulness of Tks4 and its associated molecules as biomarkers via careful statistical analyses, including Pearson’s correlation analysis, principal component analysis (PCA), multiple logistic regression, confusion matrix analysis, and ROC analysis.

**Results:**

Our findings indicate that the co-expression patterns of the seven examined biomarker candidates better differentiate between tumor and normal samples compared with the expression levels of the individual genes. Moreover, variable importance analysis of these seven genes revealed four core genes that yield consistent results similar to the seven genes. Thus, these four core genes from the Tks4 interactome hold promise as potential combined biomarkers for colon adenocarcinoma diagnosis and prognosis.

**Conclusion:**

Our proposed biomarker set from the Tks4 interactome shows promising sensitivity and specificity, aiding in colon cancer prevention and diagnosis.

## 1 Introduction

The SH3PXD2B gene encodes the Tks4 protein (tyrosine kinase substrate with four SRC homology 3 [SH3] domains), characterized by its possession of four SH3 domains and one PX domain (Buschman et al., 2009). Belonging to the scaffold protein family, Tks4 is functioning as a key podosome and invadopodia organizer and regulates the assembly of cell motility related signaling complexes. These scaffold proteins play an active role in facilitating communication among signaling molecules through their simultaneous binding (Buday and Tompa, 2010). The significance of the Tks4 gene is underscored by its association with Frank-Ter Haar syndrome, a rare developmental disorder resulting from inactivating mutations (Iqbal et al., 2010). Additionally, Tks4’s involvement in downstream EGFR signaling has long been recognized. Upon EGFR activation by EGF, Tks4 is recruited to the plasma membrane, where it undergoes tyrosine phosphorylation by SRC, thereby facilitating the association of downstream signaling molecules (Bögel et al., 2012; Dülk et al., 2018).

Tks4’s involvement in tumor formation and progression across various human cancers, including melanoma (Iizuka et al., 2016), gastric carcinoma (Zhu et al., 2023), and hepatocellular carcinoma (Kui et al., 2021), has been well documented in the literature. Our group has previously demonstrated significant epithelial-mesenchymal transition-like (EMT) morphological, functional, and molecular changes regulated by Tks4 in the HCT-116 colon carcinoma cell line (Szeder et al., 2019). During EMT, differentiated epithelial cells undergo various molecular changes leading to a mesenchymal cell type with enhanced migratory and invasive properties. These changes involve altered expression and activation of transcription factors, cell adhesion proteins, cytoskeletal proteins, extracellular matrix degrading enzymes, and others (Kalluri and Weinberg, 2009; Yeung and Yang, 2017). In a recent study, a Tks4 knockout HCT-116 cell line was investigated, revealing through transcriptome analysis the involvement of not only protein-coding genes but also several long non-coding RNAs in the EMT process modulated by Tks4 in colon cancer. These findings suggest that Tks4 plays a multifaceted regulatory role among different signaling cascades beyond its role in invadopodia formation (Jacksi et al., 2023).

**FIGURE 1:**
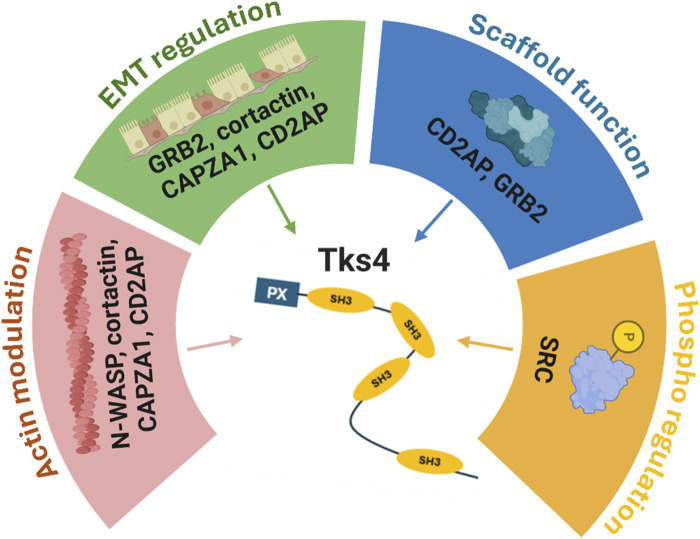
Overview of the known effects of the Tks4 interactome. A schematic representation of the protein-protein interaction network of the Tks4 scaffold protein. N-WASP, cortactin, CAPZA1, and CD2AP molecules are associated with actin cytoskeleton reorganization. Among these proteins, cortactin, CAPZA1, and CD2AP were also identified as regulators of EMT processes in cancer cells along with GRB2. Regarding the molecular function of the Tks4-associated proteins, CD2AP and GRB2 represent scaffold proteins, while SRC is a protein kinase that regulates the phosphorylation status of Tks4 itself and several Tks4-interacting proteins. *Created with BioRender.com.*

While numerous experimental findings highlight Tks4’s key role in cancer pathogenesis, limited attention has been directed towards exploring its expression patterns and assessing its potential as a tumor biomarker. Colorectal carcinoma (CRC) stands out as one of the most prevalent oncological diseases, constituting 10% of global cancer incidence with 1.93 million newly diagnosed cases (Xi and Xu, 2021). Despite the availability of screening tests such as stool tests, virtual colonoscopy, colonoscopy, and sigmoidoscopy (Lin et al., 2021), there remains a pressing need to discover additional minimally invasive methods for timely detection of precancerous conditions and accurate diagnosis of various tumor stages. Such advancements are necessary for enhancing the efficacy of personalized therapies and mitigating mortality rates. Consequently, our focus is on investigating the functionally interconnected network of Tks4-associated partner molecules within the context of colon cancer (see Figure 1).

Within the intricate network of Tks4 interacting partner molecules, Neural Wiskott-Aldrich syndrome protein (N-WASP) has a crucial role, as its allosteric activation process mediated by Tks4 initiates new actin branch formations during cellular movements (Kropyvko, 2015; Okruta et al., 2015). However, the role of N-WASP (gene name: WASL) in intestinal carcinogenesis remains controversial, with evidence suggesting it may function as a tumor suppressor in early tumorigenesis but exert a pro-invasive role in later metastatic phases (Morris et al., 2018; Yan et al., 2020). Additionally, Cortactin (CTTN), another prominent member of the Tks4-interactome, acts as a scaffold protein that colocalizes with Tks4, facilitating the assembly of multiple partners at sites of actin polymerization, including the Arp 2/3 complex, F-actin, SRC, and N-WASP (Lányi et al., 2011). Studies both *in vitro* and *in vivo* have revealed Cortactin’s role in promoting proliferation and enhancing the MAPK signaling pathway by downregulating EGF receptors in colorectal cells (Zhang et al., 2016).

Several recently identified Tks4 partner proteins play pivotal roles in cancer progression. Among them, the F-actin capping protein α1 subunit (CAPZA1) stands out, binding to and capping the barbed ends of actin filaments, thereby regulating actin assembly and cell movements (Qin et al., 2023; László et al., 2024). In hepatocellular carcinoma, CAPZA1 has been implicated in regulating actin cytoskeleton remodeling and influencing the EMT process during cell migration (Huang et al., 2017). Notably, CAPZA1 demonstrates strong immunoreactivity in malignant colorectal cells compared to normal cells, consistent with findings in cancer patient-derived tissues (Gemoll et al., 2011). Furthermore, our research group identified CD2-associated protein (CD2AP) as a protein-protein interaction partner of Tks4 in colon cancer cells (Kurilla et al., 2023). CD2AP, an actin-stabilizing scaffold protein, plays a crucial role in dynamic cytoskeleton assembly and remodeling by directly binding to filamentous actin, cortactin, Tks4, or CAPZA1. Low CD2AP expression has been associated with poor prognosis in gastric cancer and renal clear cell carcinoma patients, and its reduction *in vitro* triggers unfavorable partial EMT processes in colon cancer cells (Xie et al., 2020; Kurilla et al., 2023; Chen et al., 2024). Additionally, growth factor receptor binding protein 2 (GRB2), a long-described binding partner of Tks4, plays a significant role in cancer signaling pathways. As an adaptor protein, GRB2 participates in the regulation of the cell cycle, actin-based cell motility, and receptor tyrosine kinase signaling (Giubellino et al., 2008; Bisson et al., 2011). GRB2 is integral to the EGFR/MAPK pathway, deregulation of which has been linked to colorectal cancer formation (Koveitypour et al., 2019).

The final interaction partner scrutinized was SRC, a non-receptor tyrosine kinase well known for its direct interaction with Tks4. SRC plays a central role in regulating various cellular processes, including proliferation, differentiation, cell-cell communication, adhesion, and migration, all orchestrated through the EGF signaling pathway (Dülk et al., 2018; Kudlik et al., 2020). Notably, SRC’s significant involvement in colorectal carcinoma (CRC) pathogenesis is underscored by higher expression levels observed in CRC patient samples compared with normal mucosa. Moreover, extrahepatic colorectal metastases exhibit more than a tenfold upregulation in SRC expression levels relative to normal mucosa (Jin, 2020). The interaction between EGFR and Tks4 emerges as a critical step in SRC recruitment and activation (Dülk et al., 2018). Given the pivotal roles of SRC and EGFR as oncogenes impacting colon cancer development, exploring Tks4’s role in colon cancer becomes imperative for a comprehensive understanding of the pathomechanisms underlying colon cancer formation.

Our study aimed to provide a new interactome-based approach for biomarker development for colon cancer diagnosis based on mRNA expression data from colon cancer patient-derived tissue samples and databases. Focusing on known protein interaction networks (Figure 1), we identified specific gene sets among the Tks4 interactome that are highly sensitive and specific combined biomarkers. Furthermore, based on the known tumor-regulating function of this interactome, we hypothesized that Tks4-organized association of molecules and their expression patterns might represent a potential biomarker profile for colon cancer.

## 2 Results

### 2.1 Assessing the impact of Tks4 scaffold protein expression levels across different cancer types

**FIGURE 2:**
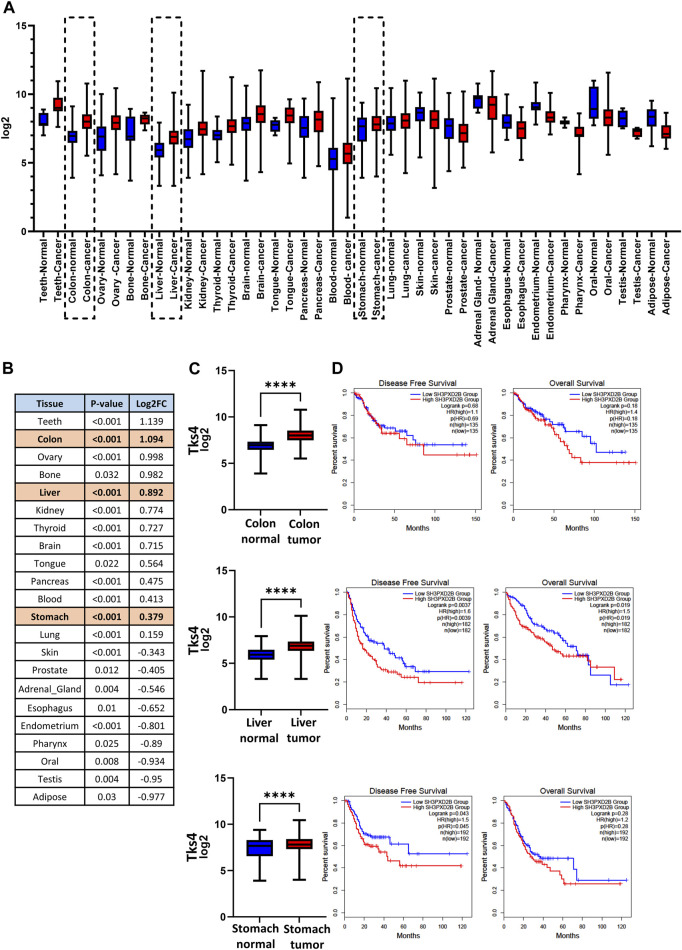
Pan-cancer analysis of Tks4. **(A)** Overview of significant Tks4 expression level differences between normal and tumor samples in different cancer types based on the Gent2 database. The further examined three cancer types are highlighted with dashed lines. **(B)** Statistical table of different tumor types based on the Gent2 database in descending order of Log_2_FC. The further examined three cancer types are highlighted with a shaded background. **(C)** Tks4 expression levels in colon, liver, and stomach normal and tumorous tissues. **(D)** Disease free survival and overall survival analysis based on low and high Tks4 expression profiles.

First, to more deeply study the Tks4 expression profiles of different cancer types, we screened large cancer patient cohorts using open-access data from The Cancer Genome Atlas project (TCGA) database. We assessed the level of Tks4 expression between cancers and their paired normal samples using the GENT2 (NCBI GEO based) database. In several cancer types, the analysis showed that the Tks4 expression level was significantly higher or lower in cancerous tissue compared with normal tissue (Figure 2A). Next, we sorted the statistically significant Tks4 Log_2_ fold changes (Log_2_FC) by each cancer type and ranked them in order of decreasing values (Figure 2B). In addition to the epidemiologically important colon cancer, which had the second largest Log_2_FC value with strong significance, stomach and liver cancers are also marked in Figure 2B because the Tks4 expression level has already been investigated as a potential biomarker in these malignancies.

We also analyzed the prognostic power of Tks4 expression on the survival of patients with these three highly statistically significant and epidemiologically important cancers. In the Gepia database the COAD samples were stratified into high and low Tks4 expression groups whose dividing point was the median cutoff point and then Kaplan-Meier curves were generated to represent the Disease Free Survival and Overall Survival of the two patient groups for each cancer type (Figure 2C). High Tks4 expression was generally associated with poorer survival in all three cases.

These results indicated Tks4 levels are significantly different in some tumor types compared with those in normal tissues and that these variations in gene expression are related to differences in disease outcome. Thus, Tks4 expression level might represent a potential biomarker not only for the previously studied tumor types (i.e., liver and gastric cancer) but also for colon cancer.

### 2.2 Tks4-interactome analysis from the perspective of biomarker research

**FIGURE 3:**
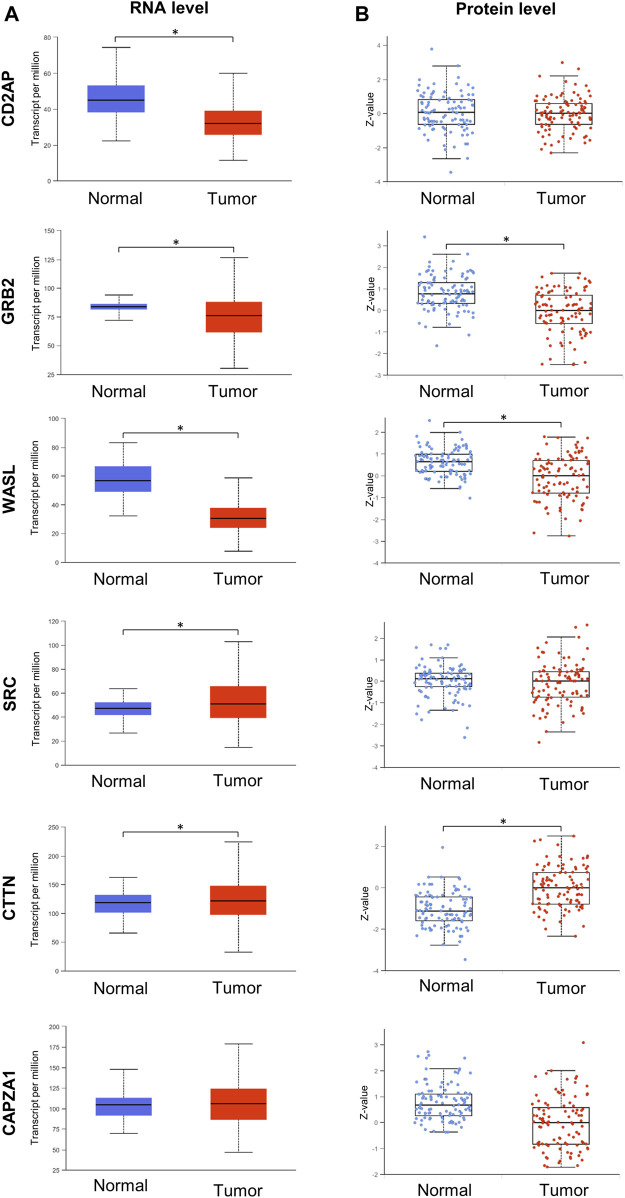
Gene expression analysis of Tks4 partner molecules. Transcripts per million mRNA copies and the Z-value of protein levels for CD2AP, GRB2, WASL, SRC, CTTN, and CAPZA1. **(A)** Gene expression levels in normal (n = 41) and primary tumor (n = 286) samples in colon cancer based on the UALCAN database. **(B)** Protein levels of normal (n = 100) and primary tumor (n = 97) samples in colon cancer based on the UALCAN database.

It is known that the expression levels of interacting partners can be correlated (Ge et al., 2001; Grigoriev, 2001) thus, we hypothesized that the Tks4 expression level and those of proteins in its interactome might be co-regulated, potentially representing a prognostic indicator in colon cancer. Using the UALCAN (TCGA-based) platform, we collected the RNA and protein levels of the Tks4-binding molecules (i.e., CD2AP, GRB2, WASL, SRC, CTTN, CAPZA1). As shown in Figure 3, the expression levels of CD2AP, GRB2, WASL, and CAPZA1 are generally downregulated, while those of SRC and CTTN are upregulated in colon cancer samples compared with their levels in normal samples at the RNA and/or protein levels.

**FIGURE 4:**
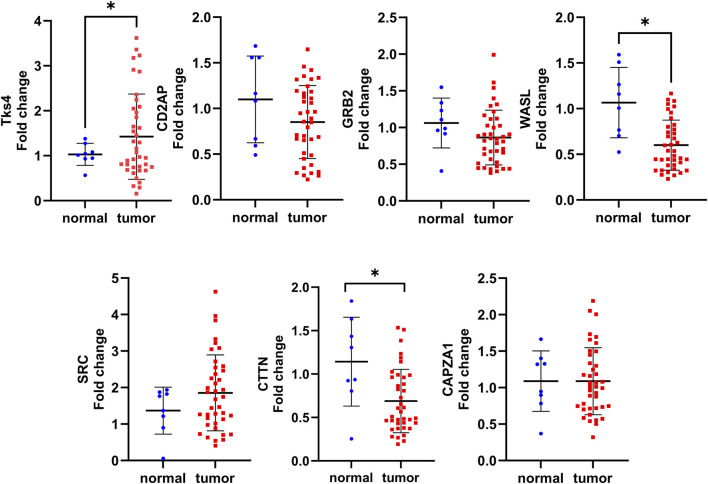
Summary of the experimental validation of the expression analysis of Tks4 and associated partner proteins. An Origene HCRT 304 human colon cancer tissue array (normal n = 8, patient derived cancer samples n = 40) was applied in qPCR measurements to quantify the expression levels of Tks4, CD2AP, GRB2, WASL, SRC, CTTN, and CAPZA1.

Importantly, the TCGA database is based on GeneChip and RNA seq method data sets, while in clinical practice, biomarker expression levels are generally measured using cheaper and specific single-gene-optimized PCR-based methods (Pang et al., 2019; Imyanitov and Kuligina, 2021). Therefore, to reliably demonstrate the usefulness of the expression levels of Tks4 and its associated molecules as biomarkers in colon cancer, we performed expression level measurements using such a method. Here, we optimized a real-time qPCR assay for each gene using commercially available primer pairs and then measured the expression levels of the Tks4 interactome in uniformly processed healthy and colon cancerous samples. To this end, we used an array on which cDNA for eight normal tissue samples and forty tumorous colon tissue samples was seeded. The use of this cDNA array allowed us to simultaneously determine the expression levels of the various genes in the same patient-derived samples, thus allowing us to assess co-expression and correlations between the analyzed genes in each sample. The results of the qPCR measurement (Figure 4) show that the Tks4 and the WASL expression levels were significantly increased and decreased, respectively, in colon cancer samples, consistent with the database analysis results (Figure 3A). The CD2AP, GRB2, SRC, and CAPZA1 expression levels did not show statistically significant increases or decreases in this relatively small dataset. The qPCR results for CTTN showed a statistically lower expression level in colon cancer samples compared to the normal samples, which is contrary to our TCGA database analysis.

We also examined the colon cancer stage-specific expression level changes of each gene examined in our qPCR measurements and compared them with the data in the TCGA database. This analysis showed that the expression level of WASL gradually decreases, while the expression levels of SRC and Tks4 gradually increase with stage (Supplementary Figure S1). The expression levels of other Tks4-interacting proteins showed trends consistent with those in the database.

Based on these results, we hypothesize that while separate measurements of each gene expression level might be predictive markers for colon cancer to some extent, a much more accurate and reliable predictive value could be achieved by considering the expression levels in combination, as a comprehensive gene expression panel.

### 2.3 Correlation analysis of the expression levels of Tks4 and its interacting proteins

**FIGURE 5:**
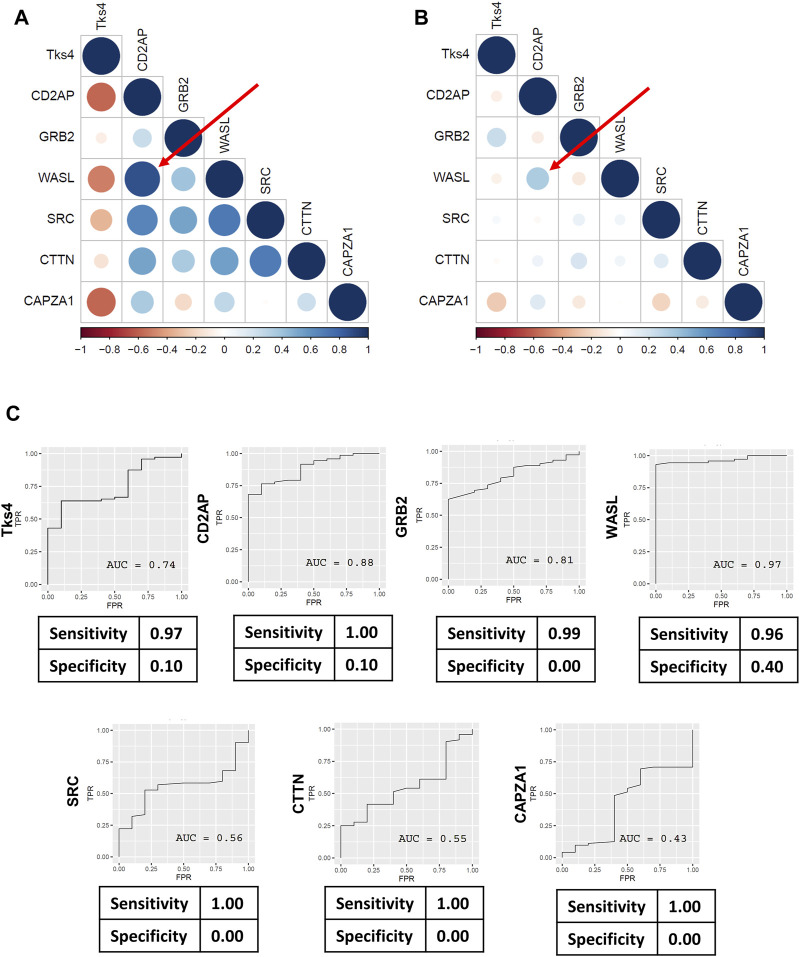
Pearson’s correlation coefficients were calculated for Tks4, CD2AP, GRB2, WASL, SRC, CTTN, and CAPZA1 genes to demonstrate the co-expression patterns within the Tks4-interactome. Heatmaps generated using **(A)** the TCGA COAD normal sample data and **(B)** the TCGA COAD tumor sample data showing the correlations between the expression levels of the seven genes. Numbers range from −1 to 1 are Pearson’s rank correlation coefficients, while the size of the circles indicate the strength of the correlation. Red represents negative correlations and blue represents positive correlations between the gene pairs. The top correlated genes are WASL and CD2AP, highlighted with a red arrow on both heatmaps. **(C)** ROC analysis results for the expression levels of each gene in the COAD patient samples from the TCGA database.

We further dissected the gene expression data from the TCGA database by using Pearson correlation analysis to evaluate the correlations between the expression levels in each gene pair, presented as co-expression matrices in Figures 5A, B. The correlation profiles were generated based on the normal and tumor samples from the TCGA COAD dataset. We found that the correlation were generally reduced between gene pairs of the tumorous samples. In contrast to this, the expression levels of the gene pairs showed high correlation in the normal samples. We then analyzed the predictive value of each gene’s expression level to better assess their usefulness for distinguishing between normal and tumorous samples. The sensitivity values gleaned from each receiver operating characteristics (ROC) analysis were generally high; however, the specificity values were very low (Figure 5C).

### 2.4 Developing a Tks4 interaction network-based biomarker prediction panel for colon cancer

**FIGURE 6:**
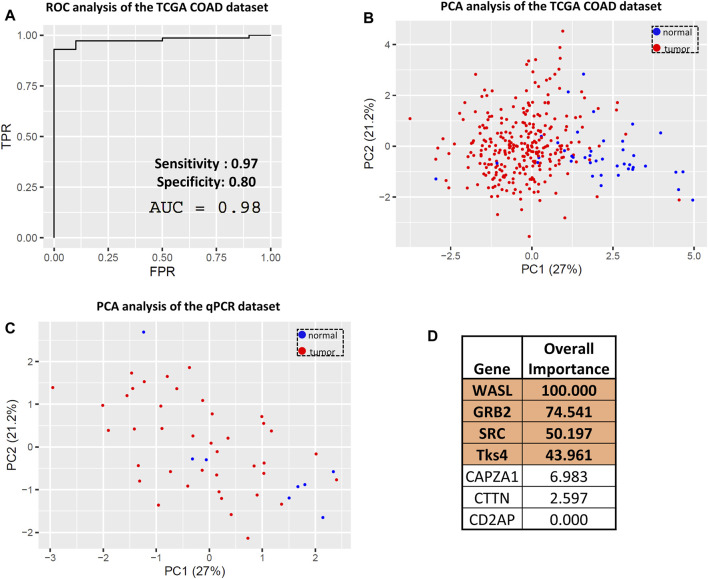
Evaluating the predictive value of the Tks4 interactome expression levels. **(A)** Combined ROC and **(B)** principal component analysis (PCA) of the TCGA COAD dataset based on the selected gene set. **(C)** Principal component analysis of the qPCR dataset based on the selected gene set. **(D)** Variable importance status of each analyzed gene. The four most important genes (WASL, GRB2, SRC, and Tks4) are highlighted.

The above results led us to analyze the expression levels of the seven genes in a combined manner and to use this dataset as a novel biomarker set for colon cancer. We constructed a data matrix comprising 41 healthy and 288 colon cancer samples using the TCGA COAD dataset from UCSC Xena and performed combined ROC and confusion matrix analyses based on a multiple logistic regression model of the expression levels of the seven genes. The area under the curve (AUC) value from this ROC analysis was 0.98, representing a promising true positive and false positive rate (Figure 6A). Consistently, the confusion matrix statistics showed that the normal and tumor samples can be reliably separated using gene expression data for CD2AP, GRB2, WASL, SRC, CTTN, CAPZA1, and Tks4 with 97% sensitivity and 80% specificity (Supplementary Figure S2; Figure 6A). These results suggest that the expression levels of the seven interacting proteins could potentially serve as a biomarker set for colon cancer detection and diagnosis. We also conducted a PCA on the same UCSC Xena dataset. The prognostic values of the PCA can be described as a multidimensional ellipsoid that fits the dataset where each axis of the ellipsoid represents a principal component. The first two PCAs were used to visualize the distribution of the samples, which readily separated the colon cancer samples (red) and the healthy samples (blue) into clusters (Figure 6B). Next, we used the same PCA transformation on our qPCR array data for validation. The two-dimensional PCA dot plot of this data set (Figure 6C) also distinguished two clusters of samples, which represented the cancerous and the normal groups. Based on a multiple logistic regression model, the importance of each variable is reflected in the absolute value of the t-statistic. This analysis expresses how much accuracy the model loses by excluding each variable. The higher the importance value, the higher the importance of the respective variable in the model (Song et al., 2013). This analysis revealed that the expression levels of WASL, GRB2, SRC, and Tks4 were the most important variables among the entire gene set (Figure 6D); therefore, we conclude that the examination of these four genes should be sufficient for colon cancer diagnosis.

Based on the above results, a multiple logistic regression analysis and subsequent ROC and confusion matrix analyses were performed using these four genes to narrow down the list of colon cancer-relevant marker genes among the Tks4 interactome.

**FIGURE 7:**
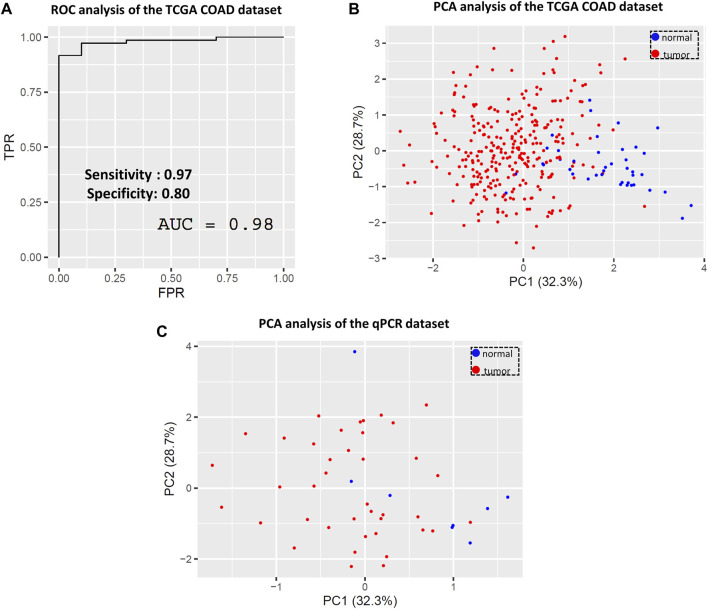
Evaluating the predictive value of the four most important genes (WASL, GRB2, SRC and Tks4) based on the variable importance analysis. **(A)** Combined ROC and **(B)** principal component analysis of the TCGA COAD dataset based on the reduced gene set. **(C)** Principal component analysis of the qPCR dataset based on the reduced gene set.

In the case of the TCGA COAD dataset, the combined ROC curve of WASL, GRB2, SRC, and Tks4 gene expression yielded the same high accuracy as before (AUC value = 0.98), indicating that the separation of normal and tumor samples is achievable based on the reduced gene set’s expression data (see Figure 7A). Subsequently, we evaluated the predictive power of these four genes using the confusion matrix statistic. Supplementary Figure S2 demonstrates that the confusion matrix, and hence the sensitivity, specificity, and predictive values, were preserved for the WASL, GRB2, SRC, and Tks4 genes model, suggesting their potential usability as a clinical diagnostic prediction panel. Additionally, we further analyzed the predictive value of the identified core set of potential colon cancer marker genes via PCA on both datasets, i.e., those collected from TCGA and generated via our in-house qPCR method. Consistent with the seven-gene-expression-based PCA, the normal and tumorous clusters are also clearly separated based on the TCGA data- (Figure 7B) and the qPCR data-dependent (Figure 7C) two-dimensional PCA dot plots from the reduced gene set. This finding reaffirms the conclusion that the combination of the WASL, GRB2, SRC, and Tks4 genes exhibits a colon cancer-specific expression profile, suggesting their suitability for use as a combined colon cancer biomarker.

After the statistical analysis of the Tks4-interacting proteins’ gene expression levels, we raised the question of whether this expression pattern is specific only to colon cancer. To address this possibility, we performed ROC and PCA analyses for the two previously mentioned cancer types, hepatocellular carcinoma and gastric carcinoma, in which the Tks4 expression level had already been investigated as a biomarker by other researchers. For these analyses, we used the TCGA LIHC and TCGA STAD datasets. We found that the sensitivity of the WASL, GRB2, SRC, and Tks4 gene expression set was high for both tumor types (0.96 and 0.99), but the specificity values were low (Supplementary Figures S3A, B). Consistent with these results, the PCA analysis did not separate the samples into two clusters (Supplementary Figures S3C, D). Based on the above analyses, we conclude that the proposed gene panel is specifically applicable for colon cancer.

**FIGURE 8:**
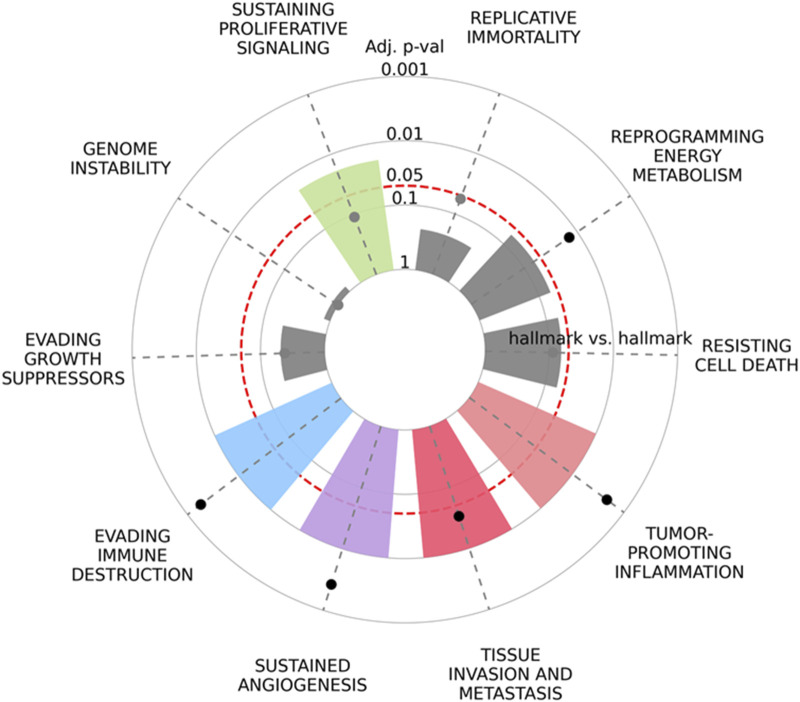
The enrichment of cancer hallmarks for the Tks4-interactome molecules CD2AP, GRB2, WASL, SRC, CTTN, CAPZA1, and Tks4 was analyzed based on https://cancerhallmarks.com. The plot illustrates the distribution and the enrichment of the gene set across the ten cancer hallmark categories.

In addition to the statistical analysis of the Tks4-interactome for its potential application as a biomarker, we aimed to provide a comprehensive understanding of the biological function of the Tks4 co-expression network in cancer formation. To achieve this, we utilized the recently released https://cancerhallmar.com database, which consolidates a consensus list of cancer hallmark defining genes from over 6,000 curated genes across various mapping resources. We entered the analyzed colon cancer marker gene set (CD2AP, GRB2, WASL, SRC, CTTN, CAPZA1, and Tks4) into this tool to elucidate which cancer hallmark-related pathways are influenced by these proteins. As shown in Figure 8, the Tks4 interactome proteins are significantly represented in pathways related to evading immune destruction, sustaining proliferative signaling, angiogenesis, tissue invasion, and metastasis cancer hallmark categories.

## 3 Materials and methods

### 3.1 Pan-cancer database analysis and GENT2 data processing

For pan-cancer analysis, we utilized expression data from the GENT2 database (http://gent2.appex.kr/gent2/) for the Tks4 gene across multiple tumor types to visualize varying expression levels. Using the results from the GENT2 database, we generated a table, ranking gene expressions based on *p*-value and log2 fold change (Log2FC) values. To investigate the expression levels of Tks4 partner molecules (WASL, SRC, Tks4, GRB2, CTTN, CD2AP, CAPZA1) at both RNA and protein levels in colon cancer, we accessed the UALCAN database (https://ualcan.path.uab.edu/index.html).

### 3.2 Survival analysis

We evaluated the correlation between Tks4 expression and disease-free survival or overall survival in the cases of colon, liver, and stomach cancer using the GEPIA2 database (http://gepia2.cancer-pku.cn/#index).

### 3.3 Colon cancer data acquisition

We obtained RNA sequencing (RNA-seq) expression data for different human cancer types from The Cancer Genome Atlas Program (TCGA) using the University of California Santa Cruz (UCSC) Xena bioinformatic tool (https://xenabrowser.net/) (Goldman et al., 2019). Specifically, we collected gene expression data for Tks4, CD2AP, GRB2, WASL, SRC, CTTN, and CAPZA1 for Colon Adenocarcinoma (COAD), Liver cancer (LIHC), and Stomach adenocarcinoma (STAD).

### 3.4 Real time qPCR measurement on tissue cDNA array

We utilized TissueScan, Colorectal Cancer cDNA IV arrays (CAT#: HCRT304, Origene) to assess the expression levels of Tks4 and its associated partner proteins at the mRNA level. The commercially available TissueScan array was in a 96 well plate format, containing 48 distinct cDNA samples sourced from human normal and cancer tissues. This array comprised eight normal tissue samples and forty cancer samples derived from various cancer stages ranging from I to IV. The classification of these stages is based on the size of the respective tumor, as well as the concurrent involvement of lymph nodes and/or distant organs (Amin et al., 2017). Notably, stage IV represents the most aggressive stage of colon cancer, characterized by metastatic cancer. The array encompassed samples from the following disease stages along with their corresponding sample numbers: stage I (n = 5), stage II (n = 9), stage III (n = 16), and stage IV (n = 10) samples. Detailed patient information regarding staging and gender is provided in Supplementary Table S1.

We performed real-time quantitative PCR (qPCR) using gene-specific primers (listed in Supplementary Table S1) and SyberGreen master mix up to a volume of 30 µL. We used the Applied Biosystems QuantStudio 5 device to measure gene expression levels for Tks4, CD2AP, CTTN, SRC, CAPZA1, WASL, GRB2, and CAPZA1. Additionally, we used a double housekeeping gene set comprising GAPDH and PUM1 as controls. We applied the 2^−ΔΔCT^ method for the analysis of the measured CT values to calculate the relative expression level. The raw data is listed in Supplementary Table S1.

### 3.5 Data preprocessing

We integrated datasets from two sources, including the TCGA database and raw data from patient-derived qPCR measurement datasets. The datasets were standardized gene-wise, mean-centered, and divided by the standard deviation. We conducted outlier detection, identifying any data points that deviated by more than 6 standard deviations from the mean.

### 3.6 Multiple logistic regression model for protein-protein interaction based bioinformatic analysis

We conducted logistic regression modelling to calculate the individual potential of a given gene in predicting normal and tumor phenotypes. Utilizing confusion matrix and ROC analysis, we identified genes significantly impacting the differentiation between tumor and normal samples and quantified model performance. Confusion matrix analysis enabled the examination of sensitivity (True Positives/(True Positives + False Negatives) and specificity (True Negatives/(True Negatives + False Positives), positive predictive value (True Positives/(True Positives + False Positives) and negative predictive value (True Negatives/(True Negatives + False Negatives) of the given biomarkers in separating tumor from normal samples. Additionally, we employed multiple logistic regression analysis to evaluate the significance of Tks4-associated partner molecules and to define a reduced model based on a subset of genes, assessing its performance compared to the full model.

A multiple logistic regression model defined by the following equation was fit to the TCGA samples using the maximum likelihood method:

$$\ln\left(p/\left(1-p\right)\right)=a_{0}+b_{i}\sum g_{i}$$

where p is the probability of a sample being a tumor sample, a_0_ and b_i_ are the coefficients to fit, and g_i_ represent the gene expression value of gene i. Model performance estimation was conducted using leave-one-out cross-validation (LOO-CV). The obtained multiple logistic regression model was also used to predict whether a sample is tumor or normal in the qPCR dataset. In all cases, confusion matrices and ROC curves were compiled from the model predictions.

### 3.7 Correlation analysis

We analyzed the correlations of standardized gene expression values for Tks4, CD2AP, CTTN, SRC, CAPZA1, WASL, GRB2, and CAPZA1 in both normal and colon cancer tissues using the TCGA COAD dataset. Pearson’s correlation analysis was conducted to assess the statistical associations between the expression levels of the Tks4-interacting partner proteins.

### 3.8 Principal component analysis (PCA)

We applied PCA to assess the variance of gene expressions among normal and tumor samples. PCA was conducted on the standardized TCGA data, and the resulting transformation was then applied to the qPCR dataset.

### 3.9 Cancer hallmark analysis

We explored the potential cancer hallmarks associated with Tks4, CD2AP, CTTN, SRC, CAPZA1, WASL, GRB2, and CAPZA1 in combination using the https://cancerhallmarks.com/ website.

### 3.10 Statistical analysis

In RStudio (Posit team, 2023) we used the statistical software R (version 4.3.1) (R Core Team, 2023) using packages “caret” (Kuhn, 2008), “corrplot” (Wei and Simko, 2021), “dplyr” (Wickham et al., 2023), “ggcorrplot” (Wei and Simko, 2021), “ggplot2” (Wickham, 2016), “Markdown” (Allaire et al., 2023), “openxlsx” (Schauberger and Walker, 2022), “pROC” (Robin et al., 2011), “readr” (Wickham et al., 2024) and “tibble” (Müller and Wickham, 2023). R Markdown code is for recreating the correlation plot, multiple logistic regression and principal component analysis results is available from https://github.com/lovricsa/Tks4analysis. Analyses were conducted on two data sets; i.e., RNA-seq expression data obtained from TCGA and in-house qPCR results.

The results from the Origene HCRT304 human colon cancer array were analyzed and visualized using GraphPad Prism 10.1.2. Tks4 expression levels were compared between groups using ANOVA with Tukey *post hoc* test or between tumor and normal tissues using unpaired *t*-test with Welch’s correction. Pearson’s correlation analysis was conducted to assess the statistical associations between the expressions of Tks4, CD2AP, CTTN, SRC, CAPZA1, WASL, GRB2, and CAPZA1, and other factors of interest. Significance was defined as a *p*-value less than 0.05.

## 4 Discussion

We have developed a novel approach for biomarker gene curation to identify colon cancer, leveraging gene expression levels of various candidates from a known protein-protein interaction network. Our primary focus revolved around the Tks4 protein and its partners, including CD2AP, GRB2, WASL, SRC, CTTN, and CAPZA1. Tks4 scaffold protein plays a pivotal role in recruiting diverse effector molecules to the plasma membrane, facilitating crosstalk among different signaling pathways influencing the actin cytoskeleton rearrangements and podosome/invadopodia formation necessary for cell movements. Throughout signal transduction processes, Tks4 collaborates with its partner molecules, and the specific composition of the resulting multi-protein complex dictates the outcome of signalization, such as altered cell motility and regulation of EMT *in vitro* in colon cancer cells. Therefore, we deliberately selected partner proteins known to be implicated in different cancer formation and focused particularly within the context of colon cancer.

We conducted an analysis of the mRNA expression levels of Tks4 and its partner proteins in both tumorous and normal tissues using data from the TCGA database. Our analysis revealed significant alterations in the expression levels of all candidates in cancerous tissues at the mRNA level, except for CAPZA1 (Figure 3).

It is important to note that while the TCGA database was established using GeneChip and RNA sequencing methods, clinical practice generally relies on cost-efficient PCR-based techniques. Therefore, to reliably demonstrate the potential applicability of the tested genes as biomarkers in colon cancer, we aimed to repeat the expression level measurements using qPCR methods. Figures 3, 4 illustrate that the database and the qPCR array analyses show similar Tks4-interactome gene expression level trends except for CTTN, which showed an opposite expression change between the two datasets. The most plausible reason for this inconsistence is that, according to the TCGA dataset analysis shown in Figure 3, the standard deviation of the CTTN levels is quite high among the patient derived colon cancer tissues. This may have an impact on the results of the qPCR dataset’s limited sample size. Therefore the high standard deviation of CTTN expression levels affects the marker’s reliability and is the cause of CTTN’s removal from the final core biomarker gene set determined by the variable significance analysis. Overall, we found in case of Tks4, WASL and CAPZA1 that similar results can be obtained from a smaller sample size, as expected based on the analysis of the larger database. In addition, the analysis of TCGA data alongside our qPCR array results indicated a significant deregulation of WASL in cancerous tissues compared to normal tissue. This finding suggests a potential tumor suppressive role for WASL during the development of colon cancer, as previously demonstrated by Morris et al. in an early intestinal carcinogenesis model (Morris et al., 2018). Furthermore, when comparing the expression levels of CD2AP, GRB2 and SRC using the results of the two datasets they also demonstrate comparable trends.

Moreover, we observed stable correlation patterns among different gene pairs in normal tissues, whereas these correlations were notably weakened in colon cancer tissues (see Figures 5A,B). The high correlation levels of gene pairs in normal tissues suggests that the expression of Tks4-interacting proteins is tightly regulated in non-cancerous state. The absence of the finetuning of this co-expression network might be the cause, or the consequence of cancer development. This led us to hypothesize that cancer-related events are associated with the disrupted correlation patterns and the loss of precisely regulated co-expression of these genes suggesting their potential utility in the diagnosis of colon cancer. We have discovered a very strong correlation and coregulation between WASL and CD2AP protein. It was already demonstrated that CD2AP and WASL form a protein complex within the T cell/antigen-presenting cell contact region during T cell activation, but their co-expressional regulation was not described before (Badour et al., 2003). On the other hand more research would be necessary to investigate the CD2AP and WASL co-regulated molecular events during cancer pathogenesis, which might be controlled by Tks4.

Next, we examined the predictive value of each gene of the Tks4 interaction network based on the TCGA database. ROC analyses and related confusion matrices revealed that these genes serve as highly sensitive biomarkers overall, although their individual specificity appeared to be insufficient (see Figure 5C). Notably, among them, the expression level of WASL emerged as the most reliable marker for distinguishing between colon cancer and normal tissues. However, its specificity value (0.40) remains suboptimal.

To enhance the utility of the selected genes, we further examined their combined expression values in colon cancer. Utilizing a multiple logistic regression model and related ROC and confusion matrix statistics, we found that this integrated model exhibited significantly higher specificity (0.80) while maintaining sensitivity (see Figure 6A) compared to individual gene expression levels (Figure 5C). As previously pointed out, pathway-based biomarkers are more useful and efficient than single-gene biomarkers (Su et al., 2021) and by using our combination analysis, we have also further increased the sensitivity of the predictive value of the examined gene set. On the other hand, from later clinical diagnostic and financial point of view, instead of huge gene panels it is more practical to use less but highly sensitive and specific biomarkers during patient diagnosis. In this study, through variable importance analysis, we successfully reduced the set of seven genes to four (Tks4, WASL, GRB2, and SRC) without compromising predictive value (Figure 7A). This reduction enhances the feasibility and affordability of such measurements for subsequent clinical diagnostics workflow.

We then explored whether the heightened specificity and sensitivity values of the reduced gene set are exclusive to colon cancer or could be applicable to other malignancies. We chose to test our gene set on hepatocellular carcinoma and gastric carcinoma TCGA datasets since the expression level of Tks4 had previously been suggested as a potential biomarker for these tumor types. While the detected sensitivity values were high, the specificity values were notably lower compared to the colon dataset (see Supplementary Figures S3A, B). These findings suggest that our proposed biomarker set of the Tks4 interactome is specific to colon cancer.

There is evidence suggesting that different colon polyps, with or without malignant potential, exhibit similar gene expression changes in the WASL- and SRC-encoding genes, as observed in our colon cancer-related analyses (Burgess et al., 2016). Based on these data, further investigation should be considered to confirm a similar expression pattern of the selected Tks4-interacting proteins in patient-derived colon cancer and pre-malignant tissue samples. If the expression values of different genes correlate, it would also be worthwhile to investigate whether the respective mRNA expression changes could be detected in stool samples (Barnell et al., 2019). This could represent a significant advancement in the early prediction or detection of colon cancer, as detecting mRNA level changes characteristic of tumors in stool samples could enable clinicians to establish more effective screening schedules for high-risk individuals, aiding in the prediction or timely diagnosis of colon cancer. Furthermore, based on the results of the larger number of clinical samples, it would also be possible to conclusively reduce or expand the applicable gene panel considering both the clinical and economical aspects.

In conclusion, our study employed a unique approach compared with the common biomarker analysis strategy. Rather than conducting high-throughput assays covering thousands of genes to identify novel biomarkers, we specifically focused on analyzing a Tks4-centered interaction network, whose members’ roles have already been confirmed in colon cancer. By adopting this biological perspective, we could avoid biases from genes without a specific effect on colon tumorigenesis. Consequently, we proposed a novel set of combined biomarkers with satisfactory sensitivity and specificity values. These findings have the potential to facilitate the prevention and timely diagnosis of colon cancer through non-invasive measurements.

## Data Availability

Tks4 mRNA expression levels were obtained from the GENT2 database (http://gent2.appex.kr/gent2/) for pan-cancer analysis. Prognostic analysis of Tks4 gene expression on disease free survival and overall survival of colon adenocarcinoma patients was investigated using GEPIA2 database (http://gepia2.cancer-pku.cn/#index). RNA sequencing expression data of Tks4, CD2AP, GRB2, WASL, SRC, CTTN, and CAPZA1 for colon adenocarcinoma (COAD), liver hepatocellular carcinoma (LIHC) and stomach adenocarcinoma (STAD) human cancer types were obtained from The Cancer Genome Atlas Program (TCGA) using the University of California Santa Cruz (UCSC) Xena bioinformatic tool (https://xenabrowser.net/). The TissueScan colon cancer cDNA Array IV datasets can be found in Supplementary Table 1. Part of the initial version of Supplementary Table 1 raw dataset has been deposited in preprint repository bioRxiv, accession number: 10.1101/2023.01.13.523903). R Markdown code is for recreating the correlation plot, multiple logistic regression and principal component analysis results is available from https://github.com/lovricsa/Tks4analysis.
