# Supplementary material for: Predictive value analysis of the interaction network of Tks4 scaffold protein in colon cancer
Source: Front Mol Biosci. 2024 Aug 21;11:1414805. doi: 10.3389/fmolb.2024.1414805 (PMC11371697; doi:10.3389/fmolb.2024.1414805)
Supplement: Supplementary file 2 [file Image1.pdf]

Supplementary Figures

Supplementary Figure S1

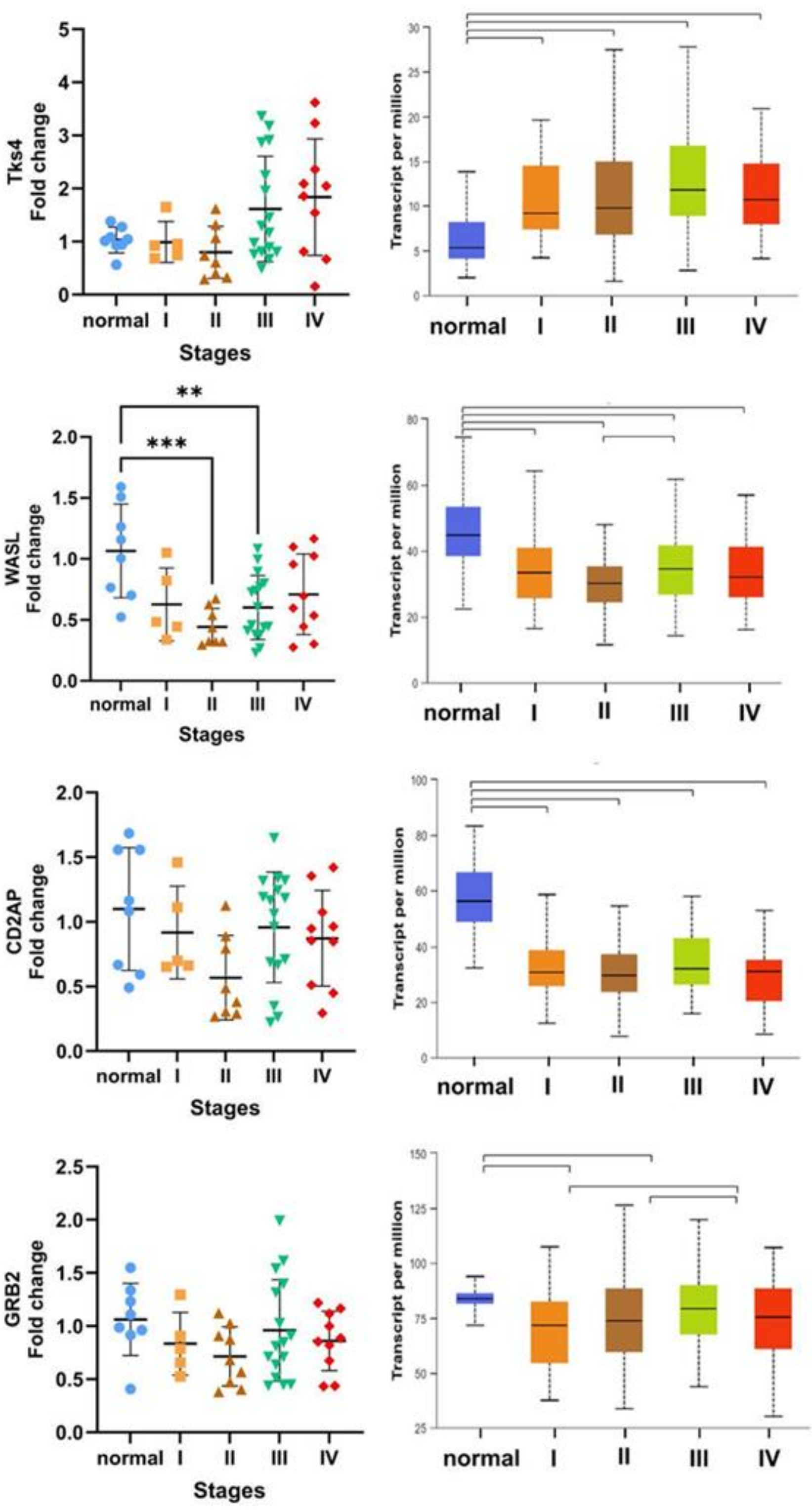

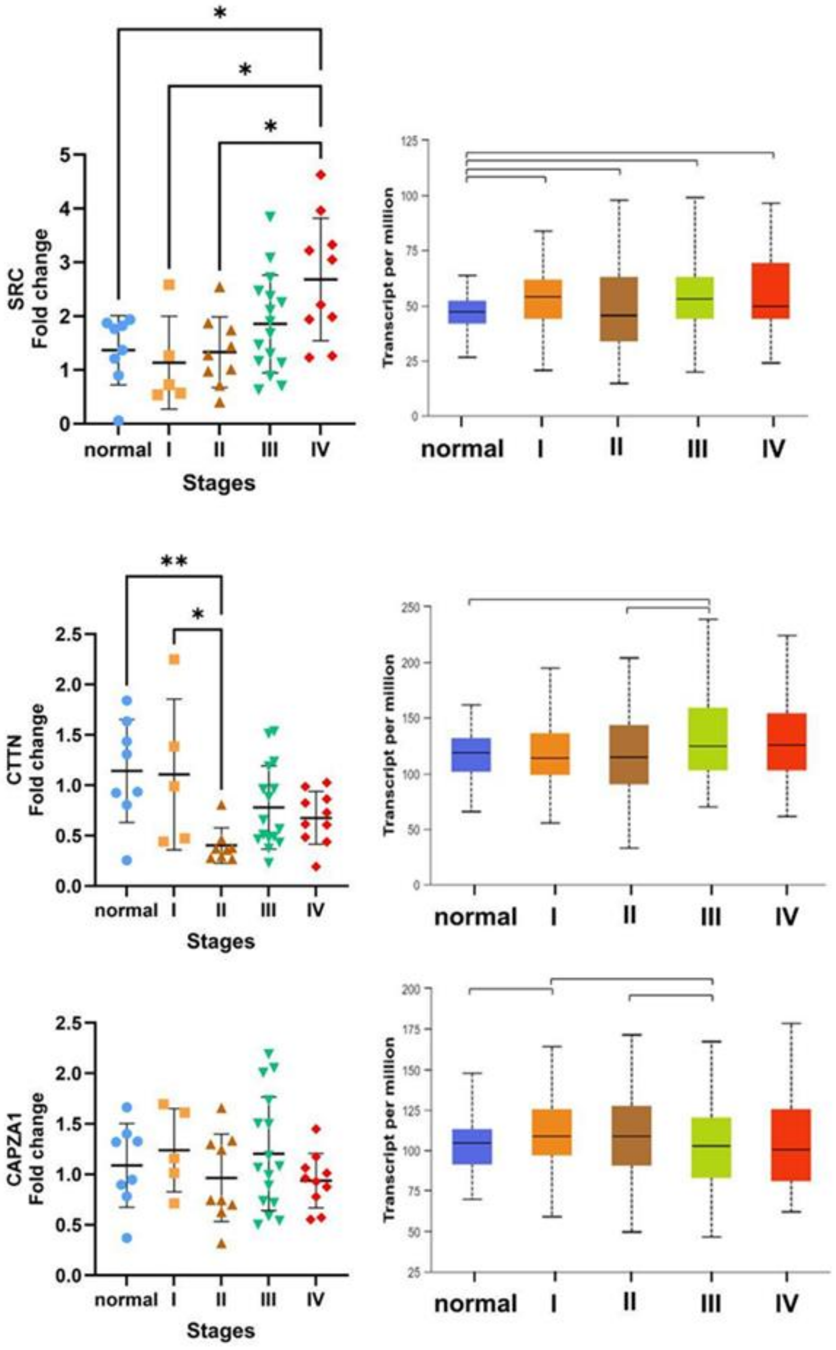

Supplementary Figure S1. Expression levels of Tks4 and its associated partner proteins in various colon cancer stages. To display the experimentally measured qPCR results and the TCGA database results separately, two different diagram types were used: experimental analysis results are represented by DotPlot diagrams (left side) and UALCAN database analysis results are represented by BoxPlot diagrams (right side). Patient numbers were as follows: the Origene HCRT 304 human colon cancer array consisted of normal (n=8), stage I (n=5), stage II (n= 9), stage III (n=16), stage IV (n=10) samples. UALCAN dataset was performed on normal (n=41), stage I (n=45), stage II (n=110), stage III (n=80), stage IV (n=39) samples. We have performed ANOVA test with Tukey's multiple comparisons on the qPCR data set. *$p < 0.05$, **$p < 0.01$, ***$p < 0.001$.

Supplementary Figure S2

A

Confusion Matrix and Statistics of TCGA COAD dataset

		Real Condition	
		Normal	Tumor
Predicted Condition	Normal	8	2
	Tumor	2	70
Sensitivity:			0.97
Specificity:			0.80
Positive Predictive Value:			0.97
Negative Predictive Value:			0.80

B

Confusion Matrix and Statistics of qPCR dataset

		Real Condition	
		Normal	Tumor
Predicted Condition	Normal	3	2
	Tumor	5	38
Sensitivity:			0.95
Specificity:			0.38
Positive Predictive Value:			0.88
Negative Predictive Value:			0.60

C

Confusion Matrix and Statistics of TCGA COAD dataset

		Real Condition	
		Normal	Tumor
Predicted Condition	Normal	8	2
	Tumor	2	70
Sensitivity:			0.97
Specificity:			0.80
Positive Predictive Value:			0.97
Negative Predictive Value:			0.80

D

Confusion Matrix and Statistics of qPCR dataset

		Real Condition	
		Normal	Tumor
Predicted Condition	Normal	4	2
	Tumor	4	38
Sensitivity:			0.95
Specificity:			0.50
Positive Predictive Value:			0.90
Negative Predictive Value:			0.67

Supplementary Figure S2. Confusion matrices for the complete gene set (CD2AP, GRB2, WASL, SRC, CTTN, CAPZA1, and Tks4) (A) using the TCGA-COAD dataset and **(B)** using the qPCR dataset. Confusion matrices for the 4 most important genes (WASL, GRB2, SRC, and Tks4) **(C)** using the TCGA-COAD dataset and **(D)** using the qPCR dataset.

Supplementary Figure S3

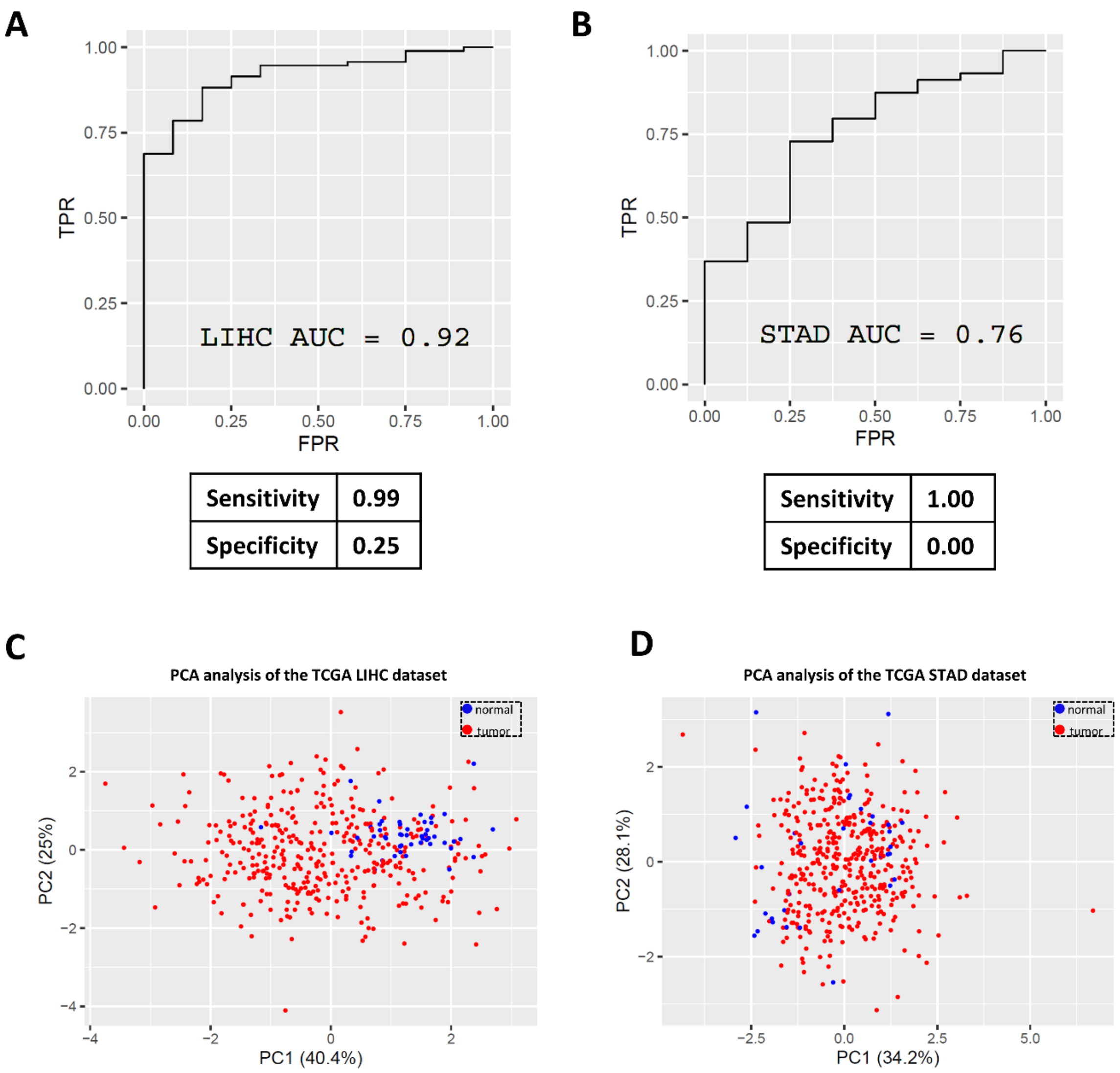

Supplementary Figure S3. Evaluating the predictive value of the four most important genes based on the variable importance analysis in liver hepatocellular carcinoma and stomach adenocarcinoma. (A) Combined ROC analysis of the TCGA LIHC **(B)** and the TCGA STAD dataset. **(C)** Principal component analysis of the TCGA LIHC **(D)** and the TCGA STAD dataset.
